# Shedding of *Brucella melitensis* happens through milk macrophages in the murine model of infection

**DOI:** 10.1038/s41598-020-65760-0

**Published:** 2020-06-10

**Authors:** Wiebke Jansen, Aurore Demars, Charles Nicaise, Jacques Godfroid, Xavier de Bolle, Angéline Reboul, Sascha Al Dahouk

**Affiliations:** 1 NAmur Research Institute for LIfe Science (NARILIS), University of Namur, Rue de Bruxelles 61, 5000 Namur, Belgium; 2 Department of Arctic and Marine Biology, Faculty of Biosciences, Fisheries and Economics, UiT-The Arctic University of Norway, Hansine Hansens veg 18, 9019 Tromsø, Norway; 3 German Federal Institute for Risk Assessment, Diedersdorfer Weg 1, 12277 Berlin, Germany; 4 RWTH Aachen University Hospital, Pauwelsstraße 30, 52074 Aachen, Germany

**Keywords:** Bacterial infection, Infectious-disease diagnostics

## Abstract

Although shedding of zoonotic brucellae in milk has been demonstrated in natural hosts, these data are still missing for the standard murine infection model. We therefore analysed shedding kinetics and the niche of *B. melitensis* in murine milk. Pregnant Balb/cByJ mice were intraperitoneally infected with 10^5^ CFU of the 16 M reference strain, a 16 M mCherry mutant or a human isolate. Milk was collected over the course of lactation, and subjected to culture and immunofluorescence assays. Bacteria were also quantified in spleen and mammary glands of maternal mice and in spleen of the litter. The shedding of the three strains did not differ significantly (*p* = 0.301), ranging from log_10_ 1.5 to 4.04 CFU/ml. A total of 73% of the mice excreted *B. melitensis* into the milk with peak values at mid-lactation; up to 30 bacteria/cell were found in macrophages and neutrophils. While the bacterial counts in the spleen of lactating females confirmed a well-established infection, only 50% of the pups harboured brucellae in their spleen, including the spleen of an uninfected pup fed by an infected foster mother. In conclusion, the murine model of infection may contribute to a better understanding of the zoonotic transmission of brucellosis.

## Introduction

Brucellosis is a severe, though neglected, zoonosis with widespread distribution and global impact^1^. The consequences on public health, animal health and economy are considerable, especially in endemic developing countries, but also in developed countries where the infection is re-emerging in wildlife^2^. The causative agents are facultative intracellular Gram-negative bacteria of the genus *Brucella*, including twelve species, of which *B. melitensis* is responsible for the main burden of disease in humans. *Brucella melitensis* is mainly isolated and transmitted from sheep and goats^3^.

In the natural host, brucellae have a predilection for both female and male reproductive organs in sexually mature animals based on immune, hormonal and metabolic factors, including the availability of erythritol. This polyhydric alcohol can be found in high concentrations in placenta, mammary gland and epididymis of most host species, but less in mice and men^4^. Brucellae enter, replicate and survive in both phagocytic and non-phagocytic cells; macrophages, dendritic cells and trophoblasts are their major target cells. *Brucella* spp. actually find excellent shelter in macrophages for their spread (shuttle), while evading the immune response of the host (stealthy organism). Replication in trophoblastic cells is strongly influenced by the stage of pregnancy; it increases in late gestation, when the cells actively secrete steroid hormones^5^. The replication in trophoblasts compromises the integrity of the placenta and shapes in consequence the clinical picture, represented by abortion and reduced fertility in the natural host. Infected animals shed the organisms in high concentrations in uterine discharges after abortion or parturition, and also intermittently or continuously in the colostrum and milk^3^. Due to the shedding of brucellae in milk, *Brucella*-contaminated raw milk and unpasteurized dairy products are the most important vehicles of human infection and a considerable public health risk even in non-endemic countries^6^. In goats infected during pregnancy, Higgins *et al*. reported shedding of *B. melitensis* 16 M in milk postpartum (pp); about half of the animals were high level shedders revealing bacterial counts of 10^4^–10^7^ CFU/ml in milk and the others shed at lower concentrations with a declining trend shortly postpartum^7^. *Brucella melitensis* was isolated periodically from the milk of ewes up to three years after infection^8^. Cows shed *Brucella abortus* up to nine years after infection^9^. In infected water buffalos, *B. abortus* was recovered from milk in concentrations up to 10^4^ CFU/ml throughout the lactation period^10^. The presence of *Brucella suis* was demonstrated in the milk of pigs as well^11^. Last but not least, even human milk may contain brucellae and was proven to be contagious to infants^12,13^.

Mice are the classical infection models widely used for studying the various aspects of pathogenesis after the infection with *Brucella*, including vertical and pseudo-vertical transmission scenarios^14^. Mice develop a discoid placenta with a flat surface facing the foetus and an irregular opposite surface adjacent to the uterine wall. The foetal labyrinthine vessels constitute the interhaemal barrier with three trophoblast layers, of which the outer one is cellular and the inner two are syncytial, forming a haemochorial placenta where the foetal chorion is in direct contact with the maternal blood^15^. Due to the structural similarity to the haemochorial placenta of women, mice are the most frequently used animal model for placenta and pregnancy research in human medicine^16^. In contrast, ruminants develop a synepitheliochorial placenta type with 100 to 140 cotyledons, which attach to maternal preformed uterine caruncles to form placentomes. Foetal villous trees consist of an epithelial surface layer, the chorionic epithelium or trophoblast, and a mesenchymal core carrying branches of allantochorionic blood vessels, and interdigitate with maternal crypts. Owing to the synepitheliochorial structure, foetal and maternal vascular systems remain completely separated from each other^17^.

In contrast, the histological structure of the mammary gland is similar in most mammalian species^18^. The functional unit of the mammary gland in all of these species is the *alveolus*, a hollow sphere surrounded by an embracing blood system. Epithelial cells synthesize and secret milk apically into the alveolar lumen^19^. The alveoli open into secondary mammary tubules, which form the *mammary duct*^20^. The blood-milk barrier inhibits the leakage of milk components from the luminal side into the blood due to epithelial tight junctions. During infection, the integrity of the alveolar epithelium is compromised and therefore enables the flow of aqueous molecules, ions, water, and microbes^21^. Pathogens will find ideal conditions to multiply, once they are inside the gland. Here, they trigger immunologic responses because of the damage of mammary alveoli and ductal epithelium. Whereas contagious or environmental mastitis results from an ascending infection through the teat canal, the subclinical mastitis due to brucellosis is caused by haematogenous dissemination of the pathogen^22,23^.

Neither the shedding of *Brucella*, nor its histopathological impact on the mammary gland, nor, and most importantly, its cellular niche in milk has yet been studied in the murine model of infection. We therefore characterized the quantitative burden and the kinetics of shedding in murine milk of experimentally infected Balb/cByJ mice. Our main objectives were, (i) to quantify the shedding of *B*. *melitensis* in murine milk, (ii) to evaluate the pathology and the nature of colonization resulting from infection in mammary glands, and (iii) to identify the cellular niche (if any) of *B. melitensis* in murine milk and mammary glands.

## Results

Neither the infection with (i) the *B. melitensis* mCherry mutant strain (mCh), nor with (ii) the *B. melitensis* 16 M reference strain (16 M), nor with (iii) the human *B. melitensis* isolate (FS) resulted in changes of behaviour or food intake. In addition, body temperature was not elevated and abortions could not be recorded. Finally, five mother mice in the mCh group and seven mother mice each in the 16 M and FS group and their litters were included in the study. The experiment was terminated in accordance with the ethical protocol when the predefined end point was reached by the pups (less than 50% weight compared to the control litter). The loss of weight coincided with insufficient milk production of the dam (<10 µl per sample).

### *B. melitensis* is shed in murine milk

**Figure 1:**
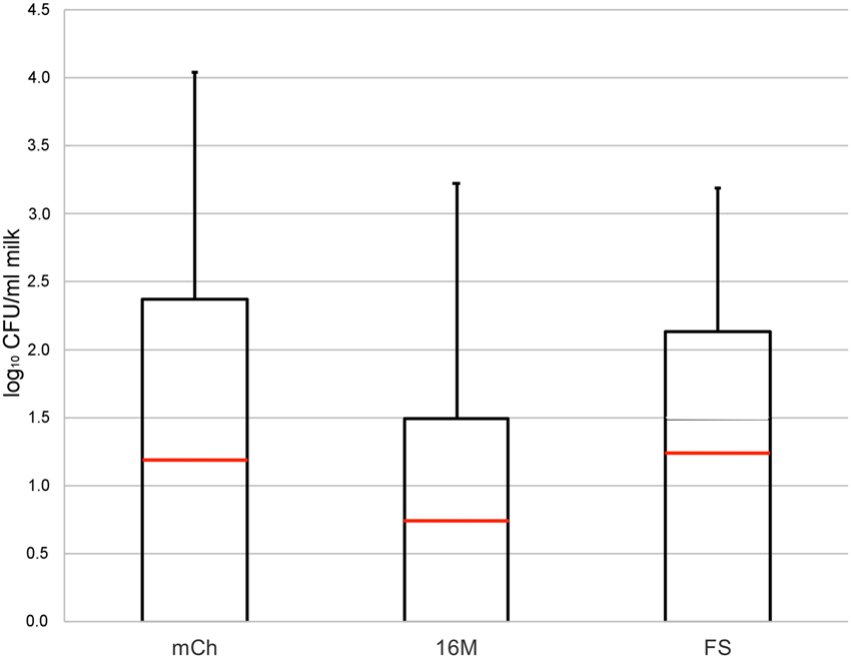
Boxplot graph (mean: red line) showing the *Brucella melitensis* counts in murine milk in log_10_ CFU/ml presented for group mCh (n = 5 mice, 27 samples), group 16 M (n = 7 mice, 27 samples) and group FS (n = 7 mice, 22 samples).

To assess the presence of *B. melitensis* in murine milk, pregnant Balb/cByJ mice were inoculated i.p. with 2 × 10^5^ CFU *B. melitensis* and milked at several points in time postpartum. In the mCh group, 80% of the mice (4/5) shed *B. melitensis* in milk, whereas in the 16 M group and in the FS group, 70% of the mice (5/7) in each group shed *B. melitensis* in milk. The quantitative results did not differ significantly among groups (*p* = 0.301) (Fig. 1). Therefore, the three group results were merged (mCh: mice no. 1–5, 16 M: mice no. 6–12, FS: mice no. 13–19, resulting in 19 mother mice altogether) and further statistical analyses were run together, regardless of the strain used for infection.

### The majority of mice shed *B. melitensis* intermittently

A total of 73% of the mice (14 out of 19) shed *B. melitensis* in their milk. The majority of mice were milked 3 to 4 times (15/19, 79%), three mice 5 to 7 times (16%) and one mouse only once (5%).

**Figure 2:**
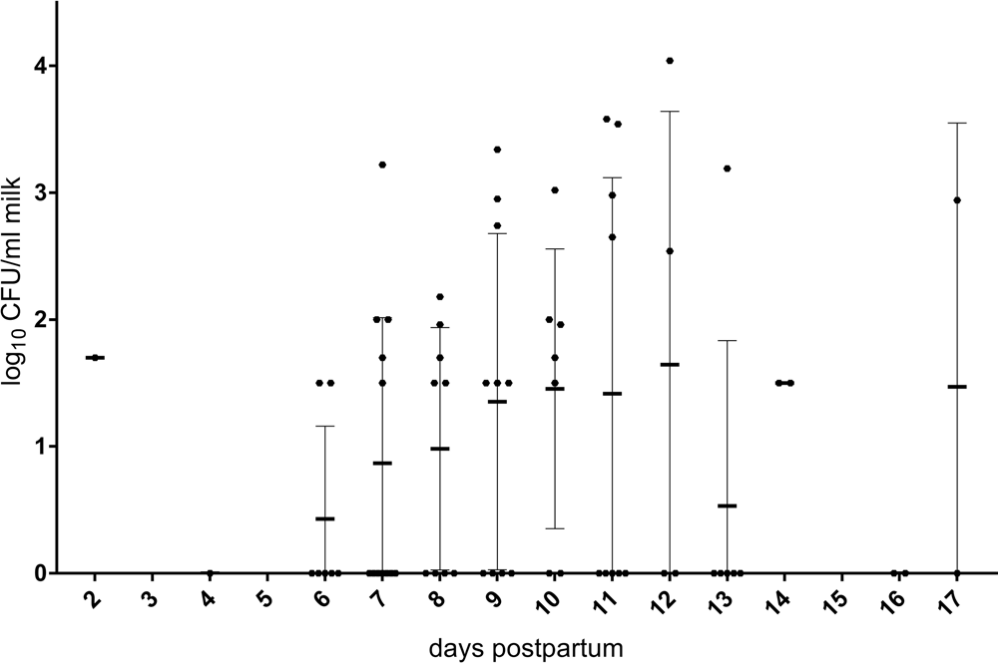
Kinetics of the shedding of *B. melitensis* in murine milk from day 2 to 17 pp.

About half of the mice (9/19) revealed bacterial shedding in milk between log_10_ 1.5 and 2.9 CFU/ml. A total of 26% of the mice (5/19; mice no. 3–5, 8 and 14) showed at least once high shedding rates (≥log_10_ 3 CFU/ml). It is noteworthy that 26% of the mice (5/19) did not excrete *Brucella* at any time during lactation. The individual kinetics usually showed intermittent shedding (79%, 11/14 mice) and merely three persistent shedders (16%) (Table 1). Taking the 76 milk samples collected from all mice together, 51.3% were negative (39/76), 39.5% (30/76) revealed a moderate contamination with *Brucella* between log_10_ 1.5 and 2.99 CFU/ml and seven milk samples (9%) exceeded log_10_ 3 CFU/ml. Positive milk samples were most frequently recorded from day 6–7 pp onwards with a maximum at day 10 pp (71.4%, 5/7 mice). The shedding rate showed the highest mean (1.65 CFU/ml) at day 12 pp (Fig. 2). However, the time effect of this qualitative and quantitative rise of the shedding comparing the seven defined sampling time points was non-significant *(p* = 0.2182).

**Table 1:** Individual kinetics, mean burden from day 1 to day 17 and shedding pattern per mouse (NEG: All samples negative, INT: Minimum of 2 samples positive and 1–4 samples negative, PER: All samples positive).

***Days pp***	***1***	***2***	***3***	***4***	***5***	***6***	***7***	***8***	***9***	***10***	***11***	***12***	***13***	***14***	***15***	***16***	***17***	***Shedding pattern***
	1			2		3		4		5		6		7				
	**log**_**10**_ **CFU/ml milk**																	
**1**						0	0									0	2.94	INT
**2**						0	0									0	0	NEG
**3**						0	2.00	0	2.74	3.02	3.54	4.04						INT
**4**		1.70		0		0	0	0	3.34									INT
**5**						0	0	1.96	1.50	1.96	3.58							INT
**6**							0		0		0		0					NEG
**7**							0		0		0		0					NEG
**8**							3.22		1.50		0		0					INT
**9**							1.70		0		2.65		0					INT
**10**							1.50		1.50		0		0					INT
**11**						1.50		1.50		0			0					INT
**12**						1.50		1.50		2.00								PER
**13**								0.00		1.70		2.54		1.50				INT
**14**							2.00		2.95		2.98		3.19					PER
**15**								1.70		0		0		1.50				INT
**16**								2.18		1.50		0						INT
**17**								0										NEG
**18**							0		0		0							NEG
**19**							1.50		2.65		2.00							PER
***MEAN***	**nd**	**1.70**	**nd**	**0**	**nd**	**0.43**	**0.92**	**0.98**	**1.47**	**1.45**	**1.48**	**1.65**	**0.46**	**1.50**	**nd**	**0**	**1.47**	
*Percentage of shedding mice (day pp)*	nd	100	nd	0	nd	28.6	46.2	55.6	63.6	71.4	55.6	50	14.3	100	nd	0	50	

^*^NEG: All samples negative; INT (intermittent shedders): Minimum of 2 samples positive and 1–4 samples negative; PER (permanent shedders): All samples positive; nd: not determined.

### *B. melitensis* was localized in murine milk macrophages and PMN

**Figure 3:**
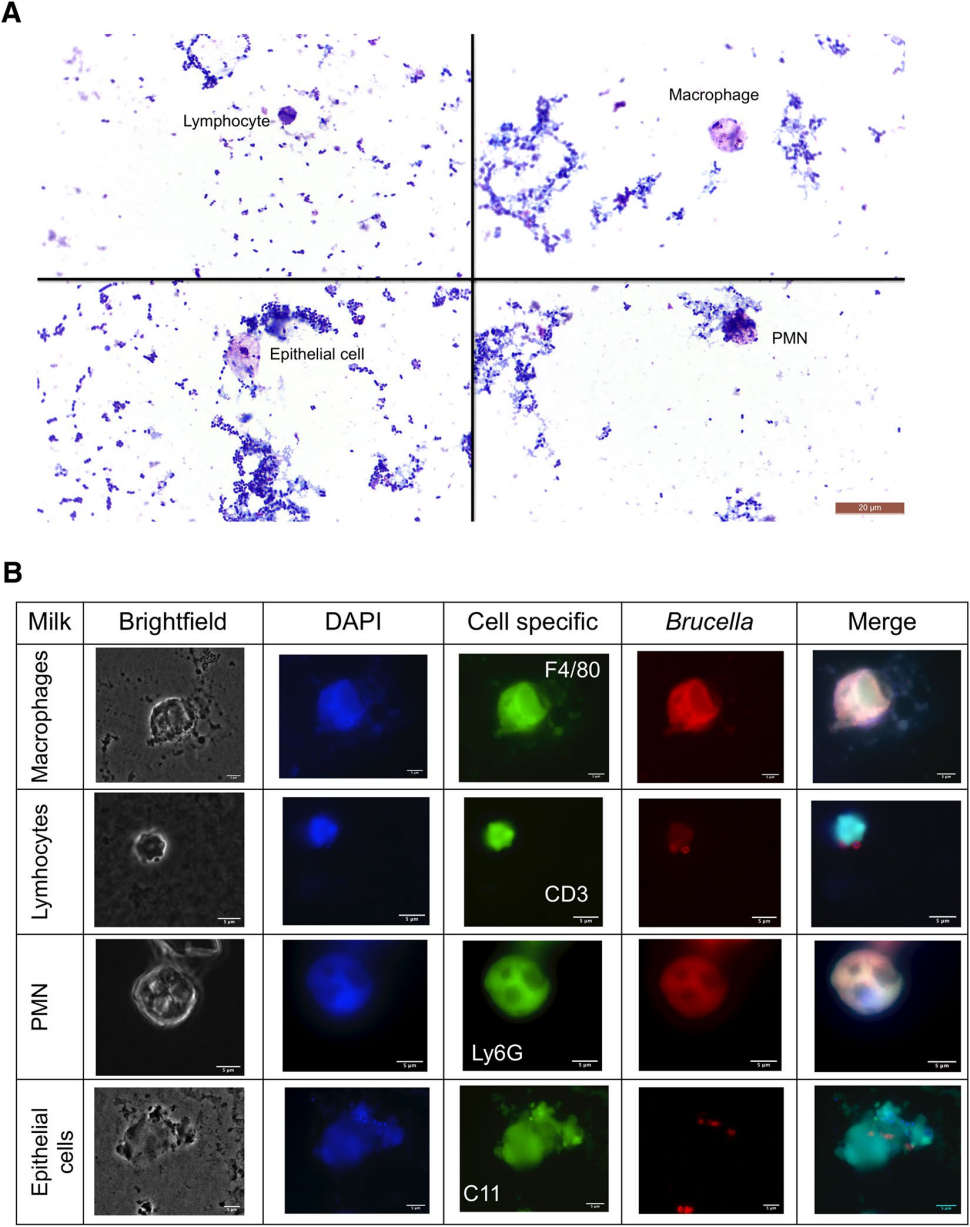
After intraperitoneal inoculation of *B. melitensis* in pregnant mice, the bacteria were found in murine milk macrophages and neutrophils. Extracellular brucellae sticked to the surface of epithelial cells. (**A**) Somatic cell counts in murine milk revealed 60% macrophages, 25% lymphocytes, 15% neutrophils, and a few epithelial cells (light microscopy, 100x oil immersion). (**B**) Milk samples were analysed using confocal microscopy for cell specific marker antigens, namely F4/80 (macrophages), CD3 (lymphocytes), Ly6G (PMN) and pan-keratin (C11, epithelial cells). The microscopic images enabled the resolution of nucleic acids stained by DAPI (blue), cell antigens (green) and *Brucella* (red).

Somatic cell counts in the milk of shedding mice were slightly increased compared to control mice and dominated by macrophages and PMN, whereas lymphocytes and epithelial cells were only sporadically found. Macrophages and PMN often contained lipid globules and amorphous globular protein (Fig. 3A). *Brucella melitensis* was mainly found in macrophages with considerable numbers up to 30 bacteria/cell and in PMN with up to 15 bacteria/cell (Fig. 3B). In contrast, brucellae were rarely found extracellularly and free-floating without association to cells in the milk. The cellular localization of *B. melitensis* did not differ between the various sampling time points.

### The mammary glands and the litter inconsistently harboured *B. melitensis*

After intraperitoneal inoculation of *B. melitensis*, bacteria were isolated from the spleen of all mice (mean: log_10_ 4.6 CFU/g). In 5 out of 19 (26%) lactating females, *B. melitensis* was detected in the mammary gland. In contrast, 9 out of 10 (90%) females that lost their entire litter due to infanticide and were sacrificed shortly postpartum (according to our study protocol) harboured *Brucella* in the mammary gland (mean: log_10_ 2.14 CFU/g (*data not shown*)). The average litter included 4 pups and about half of the pups were infected which was diagnosed by the bacterial load in the spleen. However, the prevalence of positive pups in a litter ranged from 0 to 100%. One of the infected pups originated from an uninfected foster mother of the control group but was adopted and fed for five days by an infected mother (mouse no. 17, strain FS) (Table 2).

**Table 2:** Individual burden of *B. melitensis* in murine milk, spleen, mammary gland and in the spleen of the pups/litter (mean, min, max) and day of sacrifice postpartum.

Mouse no.	Day of sacrifice pp	Mean log_10_ CFU/ml milk	Mean milk volume (µl)	Log_10_ CFU/g spleen mother	Log_10_ CFU/g mammary gland	Litter size (n)	*Brucella* positive pups (n)	*Brucella* positive pups per litter (%)	Mean log_10_ CFU/g spleen pups	Min log_10_ CFU/g spleen pups	Max log_10_ CFU/g spleen pups
1	30	0.74	58.3	3.66	na	3	3	100	3.24	2.23	4.21
2	31	0	70.8	4.55	na	3	0	0	0	0	0
3	15	2.19	37.1	4.76	2.36	7	na**	na	na	na	na
4	15	0.84	8.4	5.02	3.98	3	2	67	2.96	0	4.73
5	15	1.5	41.7	4.67	4.16	5	na**	na	na	na	na
6	16*	0	13.5	4.88	0	3	na**	na	na	na	na
7	16*	0	52.5	5.41	0	6	na**	na	na	na	na
8	15*	1.18	60	4.8	0	6	5	83	2.66	0	3.51
9	15*	1.09	66.25	4.91	0	3	3	100	3.07	3.96	4.15
10	14*	0.75	46.75	4.68	2.75	4	2	50	1.26	2	3.03
11	14*	0.75	23.25	4.34	0	7	0	0	0	0	0
12	11	1.67	56.67	4.25	0	4	2	50	1.85	2.72	1.14
13	15*	1.44	56.25	3.89	0	4	2	50	0.64	0	1.88
14	14*	2.78	42.5	4.53	0	3	3	100	3.5	3.11	3.7
15	15*	1.24	28.75	4.47	0	2	0	0	0	0	0
16	15*	0.73	45	4.96	0	4	1	25	0.77	0	3.09
17	15*	0	1	4.9	1.7	2	1	50	1.78	0	3.56
18	14*	0	19	4.35	0	4	4	100	2.51	1	3.59
19	11	2.05	33.3	na***	0	3	0	0	0	0	0
Mean			**39.6**	**4.61**	**0.88**	**4**	**1.87**	**52**	**1.62**	**1.00**	**2.44**

*According to our study protocol, mother mice were sacrificed due to the predefined endpoint of the litter (>50% weight loss compared to the control group) as a surrogate for insufficient milk production.

**Autolyzed pups were not included in the bacteriological analyses.

***The complete spleen was used for histology.

na: not available.

### *B. melitensis* was localized in resident macrophages in the mammary gland

**Figure 4:**
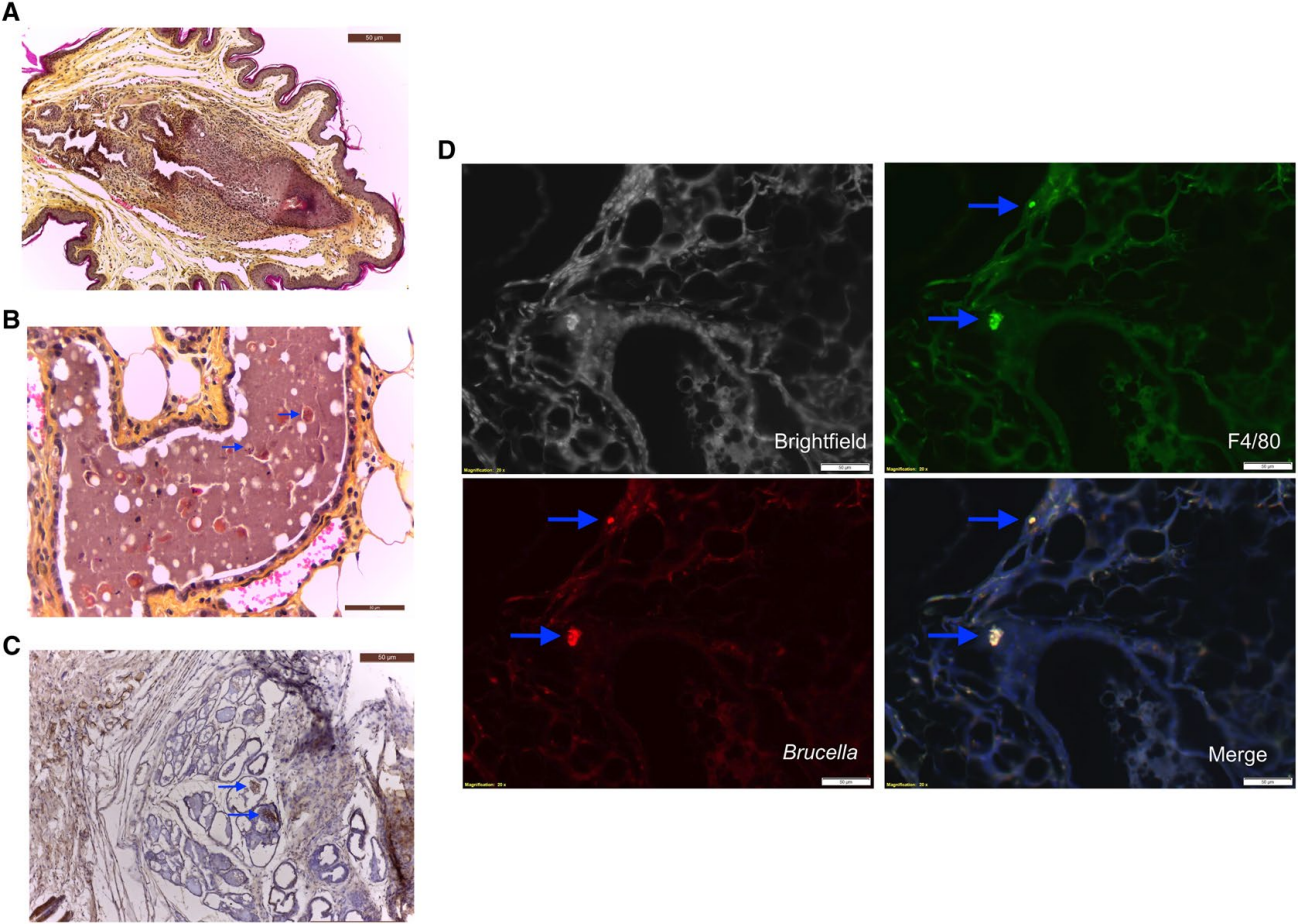
Histopathology and immunohistochemistry of murine mammary tissue infected with *B. melitensis*. (**A**) HES staining of a teat showing focal interstitial infiltration of lymphocytes, macrophages and neutrophils (10×). (**B**) HES staining of a murine mammary gland showing a lactiferous duct filled with milk, containing lymphocytes, macrophages and neutrophils as well as intracellular coccobacilli (10×) marked by blue arrows. (**C**) Immunohistochemistry of the mammary gland showing immunolabeled extracellular *B. melitensis* (10×). (**D**) Fluorescence immunolabeling of mammary tissue, analysed by confocal laser scanning microscopy, localized *Brucella* (red) in macrophages (green, F4/80).

Histologically, neutrophilic and lymphocytic cell infiltrations were observed in the interstitial tissue of the mammary glands that extended into the muscular layers resulting in a mild, subacute, interstitial mastitis. At the end of the lactation period (time of sacrifice), the mammary tissue was moderately atrophic and showed a lobular inflammation with infiltrating macrophages, lymphocytes and PMN. In case of residual milk in the alveolar lumen, coccobacilli were found in the milk macrophages. The milk duct epithelium and periductal stroma was flattened and marked by a squamous meta- and hyperplasia, and was also infiltrated with lymphocytes and macrophages. The lumen of the ducts contained PMN, lymphocytes, macrophages and cell debris. Figure 4A shows the histopathology of a mammary duct stained with HES. In the ductal lumen, intracellular coccoid bacteria were found (Fig. 4B). Furthermore, specific and localized foci of non-cell associated brucellae in the lumen of mammary ducts were visible (Fig. 4C). Immunofluorescence also showed *Brucella* in macrophages in the mammary tissue (Fig. 4D).

## Discussion

We characterized the kinetics of *B. melitensis* shedding in murine milk after experimental infection during gestation. Furthermore, we identified the cellular niche of the bacteria in the milk and evaluated histological changes in the mammary gland.

### Shedding kinetics and cellular niche of B. melitensis in murine milk

Though several studies on *Brucella* transmission already quantified the bacterial load in milk and the mammary gland of the target species, we present here the first report on the isolation and quantification of *Brucella* from murine milk in the standard model of infection. Among 19 *B*. *melitensis* infected mice, 14 (73%) shed the pathogen in their milk. About half of the mice (9/19) revealed moderate contamination loads of the milk during the lactation period, whereas 26% (5/19) of the mice were high level shedders with >log_10_ 3 CFU/ml. It was previously reported, that water buffalos naturally infected with *B*. *abortus* heterogeneously shed brucellae in milk. Most of the animals shed at low levels, but 16% of the infected buffalos were classified as “super-spreaders” since they consistently shed high *Brucella* concentrations in the milk with ≥ log_10_ 4 CFU/ml^10^.

In our infection model, we observed that more mice were shedding towards mid-lactation and the total burden of brucellae in milk peaked quantitatively at day 10 pp. In contrast, goats excrete *B. melitensis* most frequently at the beginning of the lactation period^7^. Though the kinetic trend was obvious in our study, the smaller sample sizes at the beginning and at the end of the sampling period, leading to lower volumes of murine milk, may have biased the results. Moreover, we worked exclusively with nulliparous females. In mammals, nulliparous females tend to provide less milk during their first lactation than multiparous females. Without continuous monitoring of *Brucella* concentrations in milk during subsequent lactation periods, it is difficult to say whether multiparous females still shed *Brucella* in their milk, and if so, in which quantity. In sheep, experimentally infected with a single shot of *B*. *melitensis*, shedding in milk persisted for at least three reproductive cycles post-infection and the percentage of sheep shedding *Brucella* in milk was 79%, 64%, and 38% over the first, second, and third reproductive cycle post-infection, respectively^8^.

*Brucella* was localized in murine milk macrophages and PMN, with up to 30 bacteria per cell. Macrophages derive from blood monocytes, once entering tissues they promote both innate and acquired immune responses. The innate functions of macrophages include acidification and destruction of the phagocytosed bacteria by reactive oxygen and nitrogen species^24^. *Brucella*, however, can reside in macrophages which actually should eliminate the invading bacteria. According to our understanding, *Brucella*-containing phagocytes migrate from the blood stream to the mammary alveolus due to the compromised blood-milk-barrier and subsequently into the milk. This contamination pathway was already suggested for goats^22^. PMN are the second line of immune defence in mammary gland infections; their numbers are up to 50 times higher during the first hours of mammary gland infection in bovines^25^. Similar to macrophages, PMN phagocyte and kill bacteria but PMN also phagocyte milk fat globules^22,26^. We confirmed the presence of fat globules in PMN and also in murine milk. Extracellular brucellae were seen rarely in the milk and were most likely leftovers of lysed phagocytic cells. Brucellae were not detected in lymphocytes or epithelial cells, although *Brucella* is known to persist in uterine epithelial cells^27^ and is able to replicate in murine B lymphocytes^28^.

### Histopathology and tissue burden of spleen and mammary gland

No clinical symptoms of brucellosis were observed in pregnant mice although bacteriological results revealed 100% infection of the females with high splenic tissue burden. Demars and colleagues demonstrated, that intraperitoneal inoculation results in a widely disseminated infection in mice affecting almost every organ^29^. In our study, *Brucella* could be isolated from the spleen of all mice, but the bacteriological results of the mammary glands varied. *Brucella melitensis* was merely isolated from the mammary gland of 5 out of 19 lactating mice, of which one mouse (no. 17) did not even shed the pathogen. The low isolation rate from mammary tissue of breastfeeding mice compared to the high prevalence of *Brucella*-positive mammary glands in non-breastfeeding mice (90%) suggests that the recovery of brucellae from the mammary tissue substantially depends on the residual milk volume in the gland. Meador *et al*. studied the effect of nursing on the quantity of *B. abortus* in the mammary tissue of goats^30^. Non-nursed mammary glands revealed high titres of brucellae in milk, moderate interstitial mastitis, and brucellar antigen in macrophages primarily located in alveolar and ductal lumina. In contrast, nursed mammary glands showed fewer brucellae in milk, minimal inflammatory changes, and no detectable brucellar antigen in histological sections^30^. We therefore conclude that bacterial shedding in murine milk is usually more frequent and constant than the occurrence of *Brucella* in the mammary gland or mammary lesions. It was shown that *B. abortus* was more often isolated from milk of infected cows (12/21 cows; 57%) than from the mammary tissue (8/21 cows; 38%)^23^. Histologically, the lesions in the mammary gland of mice mainly corresponded to the lesions described in target species. *Brucella abortus* caused only mild lesions in the mammary glands of goats such as multifocal to diffuse, mild interstitial mastitis with infiltration of macrophages^27,30^. This was recently confirmed in cattle^23^, showing that cows infected with *B. abortus* developed a mild interstitial inflammatory reaction in the mammary gland. However, we further observed atrophied, squamous ductal epithelium which might have been caused by the mechanical manipulations of the miniscule teats of mice during milking.

Despite of the high prevalence of *B. melitensis* in milk and mammary glands of mice, only 50% of their pups were found to be infected. Interestingly, the antibody titres we measured in the lactoserum (*data not shown*) revealed no differences regardless of the presence of *Brucella* in milk or the infectious status of the pups. The incomplete transmission of virulent *B. abortus* 544 from pregnant mice to the foetus *in utero* was already demonstrated^31^. Furthermore, vaccination reduced the amount of infected pups in this study by 50% which was linked to a placental barrier protection mediated by T- and B- lymphocytes^31^.

We recorded the infection of a primarily non-infected pup which has been suckled by an infected foster mother for five days. Bosseray *et al*. showed that the infection of infant mice, born by non-infected dams but drinking the milk from infected foster mothers, is a rare event^32^. Although the mammary glands of nursing mice were colonized with *Brucella*, less than one percent (1/114) of the pups was infected^32^. It remains to be elucidated, why the transmission to murine offspring is incomplete and which conditions finally trigger the infection of the foetus *in utero* and of the pups. Pseudo-vertical transmission through colostrum or milk^12^ and vertical transmission *in utero*^33^ may also occur in human brucellosis cases, but these kinds of transmission are rare events although placentation in mice and men is similar.

## Conclusions

Our results closed some of the knowledge gaps on brucellosis in the murine model of infection. We characterized the shedding pattern of *B. melitensis* in milk peaking in the mid-lactation period and clearly identified the cellular niche of *B. melitensis* in milk macrophages and neutrophils. The colonization of the mammary gland by *Brucella* using blood macrophages as a vehicle resulted in a mild interstitial mastitis. Even though, the research on murine brucellosis has its own value, the mouse model may also contribute to further exploration of zoonotic brucellosis mainly transmitted from animals to men through the consumption of unpasteurized dairy products.

## Material and Methods

### Ethical approval

The experimental setting and the procedures in this study were in accordance with current European legislation (Council Directive 86/609/EEC) and the corresponding Belgian law “Arrêté royal relatif à la protection des animaux d’expérience du 6 avril 2010 publié le 14 mai 2010”. The Animal Welfare Committee of the Université de Namur (Belgium) reviewed and approved the complete protocol (number of application approval: GIN 18-308). Animals were monitored daily and all experiments were performed under biosafety level 3 containment according to Council Directive 98/81/EC of 26 October 1998.

With limited food resources, the number of pups per litter declines (Weber and Olsson, 2008). We therefore improved postpartum conditions, and optimized the diet of the mothers with high protein (12.5–18%) and lipid contents (5%), supplemented with calcium and phosphorus (0.4%), vitamin A (500 IU/kg) and vitamin D (150 IU/kg). To reduce maternal stress and increase pup survival, the number of pups was limited to a maximum of 5 per litter. In case a litter included fewer pups, adoptions were carried out as described by Weber and Olsen^34^.

### Bacterial strains and mice

Wild-type SPF Balb/cByJ mice aged 8–12 weeks were purchased from Charles River Laboratories (Ecully, France). Females were synchronized three days on the bedding of males. Two females and one male were housed together at a time. After five days of mating, the male was removed. Gestation was determined manually starting from day 11. At day 14 of the gestation period, the pregnant mice were inoculated intraperitoneally (i.p.) with 2 × 10^5^ CFU *B. melitensis* in 500 μl phosphate buffered saline (Gibco PBS, ThermoFisher Scientific, Merelbeke, Belgium). In the first group, a mutant strain of *Brucella melitensis* 16 M was used, which steadily expresses the fast maturing variant of the red fluorescent protein DsRed (mCherry, mCh)^35,36^. In the second group, the unmodified *B. melitensis* 16 M reference strain (16 M) was used. In the third group, the field strain *B. melitensis* L311/15 (FS) isolated from a Turkish patient in Belgium was used^37^. Bacteria were grown in biosafety level 3 laboratory facilities overnight in 2YT media (Luria-Bertani broth with double quantity of yeast extract) shaking at 37 °C, and were washed twice in PBS (3,500 rpm, 10 min) before inoculating the mice. The control animals were inoculated with the same volume of PBS. The infectious doses were validated by plating serial dilutions of the inoculum. Those females that lost their litter due to infanticide as well as the females that resorbed the foetuses *in utero* were sacrificed for bacteriology of the spleen and mammary gland.

### Bacteriological quantification of B. melitensis in murine milk and tissue

Mice were milked, in group mCh, between day 2 and day 17 pp, in group 16 M between day 6 and day 13 pp, and in group FS between day 7 and day 14 pp according to the protocol of Gómez-Gallego *et al*.^38^ with slight modifications based on Muranishi *et al*.^39^. Briefly, females were separated from the litter for 6 h, and after exogenous oxytocin stimulation (2.5 IU i.p.), the females were anesthetized for 35–45 min with a cocktail of xylazine (10 mg/kg) and ketamine (100 mg/kg) in sterile saline solution administrated i.p. (0.1 ml/10 g body weight). Milk was collected manually and milk volume was recorded. The milking frequency was adapted from daily to every other day after the mCh group, because of the high mortality rate among pups. Moreover, the milk volume taken was limited to a maximum of 150 µl/milking. In accordance with ISO 4833-2:2013, milk samples were initially diluted tenfold in 2YT. For quantification, 100 µl of serial decimal dilutions of the milk were plated in duplicate on tryptone agar containing antibiotic supplement (*Brucella* Selective Supplement SR83; ThermoFisher Scientific) and 10% equine serum, known as Farrel’s medium. Plates were incubated for up to 12 days at 37 °C under aerobic conditions and controlled for bacterial growth every 48 h. Typically, bacterial colonies were visible within the first five days. To avoid false-negative results in the quantification process, an enrichment step was additionally performed. Remaining milk samples were incubated for 24 h at 37 °C and another 100 µl were plated on Farrel’s medium and incubated as described above. Brucellae were identified phenotypically^40^.

At the end of the experiment, females were sacrificed by double cervical dislocation. The litter was sacrificed by a lethal overdose of isoflurane (IsoFlo w/w 100% liquid, Zoetis, Zaventem, Belgium). The spleen and the second thoracic mammary complex were taken aseptically, homogenized in PBS/0.1% X100 Triton (Sigma-Aldrich, Machelen, Belgium) and plated in decimal serial dilutions in duplicates on Farrell’s medium.

### Milk cytology

The remaining milk was centrifuged at 14,000 rpm for 15 min. The supernatant was removed and the lactoserum in the intermediate phase separated for further analysis. The milk cell pellet was prepared for a Kieler sediment smear. Cells were fixed overnight at 4 °C in 4% formaldehyde at pH 7.4 (VWR, Leuven, Belgium), washed twice in PBS and re-suspended in 0.1 ml PBS. A total of 10 µl of the cell suspension was spread in a monolayer over a 3 cm^2^ field on a microscope slide^41^ and stained with Giemsa (Sigma-Aldrich, Overijse, Belgium) for light microscopy. Up to 100 cells, including macrophages, epithelial, lymphocytic and neutrophilic cells, were identified and counted at a 1000-fold magnification.

### Histology

The mammary glands were fixed in 4% formaldehyde at 4 °C for 24 h, and washed twice in PBS for 30 min. Organs were dehydrated and embedded in paraffin. Tissue sections (6 µm) were mounted on SuperFrost microscope slides (ThermoFisher Scientific). Serial sections were stained with haematoxylin eosin saffron (HES) according to standard protocols.

For immunohistochemistry and immunofluorescence staining, tissue sections were dewaxed. Immunohistochemistry was performed as described by Nicaise *et al*.^42^. In brief, after the sections had been rehydrated with PBS, endogenous peroxidase activity was inhibited with 3% hydrogen peroxide for 10 min and blocked for 1 hour using 5% normal serum. Slides were then incubated with a rabbit anti-*Brucella* serum (1/500) for 1 h at room temperature, followed by a biotinylated secondary anti-rabbit antibody (1/100, VECTASTAIN), visualized with avidin-horseradish peroxidase (1/200, VECTASTAIN) and diaminobenzidine with haematoxylin counterstaining, and finally mounted with DPX mounting medium for histology (Merck, Darmstadt, Germany). Immunofluorescence was performed on monolayer milk smears and tissue sections as described by Demars *et al*.^29^. In brief, slides were blocked for 20 min and incubated for 1 h with one of the following mAbs or reagents: FITC-coupled 1.45-2C11 (anti-CD3, eBiosciences, 1/200), FITC-coupled 1A8 (anti-Ly6G, Biolegend, 1/200), FITC-coupled Pan-Keratin (anti-C11, CellSignalling Technology, 1/200) or PE-coupled BM8 (anti-F4/80, eBiosciences, 1/200) for cells. Bacteria were stained simultaneously with a rabbit anti-*Brucella* serum (1/500). The secondary anti-rabbit antibody was coupled to Alexa-647 (Life Technologies, 1/400). Slides were mounted with ProLong Antifade containing DAPI (ThermoFisher Scientific). Labelled tissue sections were visualized with an Axiovert M200 inverted microscope (Zeiss, Jena, Germany) equipped with a high-resolution monochrome camera (AxioCam HR, Zeiss). Images (1,384 × 1,036 pixels, 0.16 μm/pixel) were taken sequentially for each fluorochrome with A-Plan 10x/0.25 N.A. and LD-Plan-NeoFluar 63x/0.75 N.A. dry objectives and recorded as eight-bit grey-level.zvi files. Three slides per organ from three different animals were analysed resulting in representative images for the experiment.

### Statistics

Quantitative data were recorded, processed and analysed in Microsoft Excel and SPSS. The non-parametric Kruskal-Wallis test was applied to detect statistical differences between the strains and their shedding in milk and tissue. The frequencies of contamination and bacterial loads per milking time point, compiled for milking day 1–3 pp (time point 1), day 4–5 pp (time point 2), day 6–7 pp (time point 3), day 8–9 pp (time point 4), day 10–11 pp (time point 5), day 12–13 pp (time point 6) and day 14–17 pp (time point 7), were analysed by the FREQ procedure and subjected to a Jonckheere-Terpstra test for associations between pathogen load and time point of milking using a significance level of 0.05. Data were plotted in Excel and Prism GraphPad.
